# Presence of Periodontopathic Bacteria DNA in Atheromatous Plaques from Coronary and Carotid Arteries

**DOI:** 10.1155/2015/825397

**Published:** 2015-10-04

**Authors:** Malgorzata Szulc, Wojciech Kustrzycki, Dariusz Janczak, Dagmara Michalowska, Dagmara Baczynska, Malgorzata Radwan-Oczko

**Affiliations:** ^1^Department of Periodontology, Wroctaw Medical University, Ulica Krakowska 26, 50-425 Wroctaw, Poland; ^2^Department of Cardiac Surgery, Wroctaw Medical University, Ulica Borowska 213, 50-556 Wrocław, Poland; ^3^Department of Vascular, General and Transplantation Surgery, Wroctaw Medical University, Ulica Borowska 213, 50-556 Wrocław, Poland; ^4^Department of Forensic Medicine, Molecular Techniques Unit, Wroctaw Medical University, Ulica M. Curie-Skłodowskiej 52, 50-369 Wrocław, Poland; ^5^Division of Oral Pathology, Department of Periodontology, Wroctaw Medical University, Ulica Krakowska 26, 50-425 Wroctaw, Poland

## Abstract

*Objectives*. Interest in periodontitis as a potential risk factor for atherosclerosis and its complications resulted from the fact that the global prevalence of periodontal diseases is significant and periodontitis may induce a chronic inflammatory response. Many studies have analyzed the potential impact of the *Porphyromonas gingivalis*, major pathogen of periodontitis, on general health. The purpose of this study was to find the presence of the *Porphyromonas gingivalis* DNA in the atherosclerotic plaques of coronary and carotid arteries and in the periodontal pockets in patients with chronic periodontitis, who underwent surgery because of vascular diseases. *Methods and Results*. The study population consisted of 91 patients with coronary artery disease or scheduled for carotid endarterectomy. The presence of *Porphyromonas gingivalis* DNA in atheromatous plaques and in subgingival samples was determined by PCR. Bacterial DNA was found in 21 of 91 (23%) samples taken from vessels and in 47 of 63 (74.6%) samples from periodontal pockets. *Conclusions*. *Porphyromonas gingivalis* DNA is frequently found in atheromatous plaques of patients with periodontitis. That is why more research should be conducted to prove if this periopathogen may have an impact on endothelium of patients at risk of atherosclerosis.

## 1. Introduction

Research over the past two decades has suggested that, beyond the conventional risk factors leading to the development and progression of atherosclerotic plaques, the condition may also result from microorganisms, increased levels of fibrinogen, the level of the white blood cell count, C-reactive proteins, and antibodies directed against heat shock proteins HSP 60.

On this basis, the immunoinflammatory theory of atherosclerosis was established, according to which atherosclerosis is a chronic immunofibroproliferative inflammatory response to factors that damage vascular endothelial cells [1]. Significant evidence proving the participation of chronic inflammation in the pathogenesis of atherosclerosis and the destabilization of existing atheromatous plaques in the arteries has led many researchers to focus their attention on searching for the cause of the inflammation. Many bacterial species have been suspected of playing an important (potential) role in atheroma development. The presence of the DNA of several microorganisms in atherosclerotic plaques, for example,* Chlamydia pneumoniae*,* Helicobacter pylori, Cytomegalovirus (CMV)*, and HSV, has been reported [2–5].

Interest in periodontitis as a potential risk factor for atherosclerosis and its complications resulted from the fact that the global prevalence of periodontal diseases is extremely high and moreover, periodontitis may induce a chronic inflammatory response. Mild forms of periodontal diseases, including gingivitis, affect up to 70% of the general population, whereas approximately 10% to 15% of the population have a more severe process with destruction of the tooth-supporting tissues [6]. Many studies since 1989 have analyzed the potential impact of the* Porphyromonas gingivalis*, major pathogen of periodontitis, on general health and especially cardiovascular problems [7, 8]. Epidemiological studies have focused on proving this relationship. In patients suffering from periodontitis with damaged periodontal pocket epithelium, transient bacteremia not only occurs during and after medical procedures such as scaling and root planning, but also may be caused by toothbrushing and even simple mastication [9].

Some data show that periodontitis is significantly associated with biomarkers of endothelial dysfunction and dyslipidemia [10]. Other authors show that* Porphyromonas gingivalis* induces foam cell formation in murine macrophage cell cultures in the presence of LDL [11].

Experimental evidence has been provided in animal models. Li et al. [12] investigated the effect of repeated systemic inoculations with* Porphyromonas gingivalis* on the progression of atherosclerosis in heterozygous apolipoprotein E-deficient (ApoE(+/−)) mice. Lalla et al. [13] assessed the impact of oral inoculation with the* Porphyromonas gingivalis* on atherogenesis in hypercholesterolemic apolipoprotein E-null mice. Both authors concluded that long-term challenge with* Porphyromonas gingivalis* can accelerate atherogenic plaque progression.

Action of* P. gingivalis* is mediated through many virulence factors, such as gingipains, hemagglutinins, fimbriae, and lipopolysaccharides (Pg-LPS). Therefore it not only becomes destructive for periodontal tissues, but also can induce and enhance general inflammation [14].

The purpose of the present study was to assess the status of periodontal tissues in patients scheduled for surgery due to atherosclerosis and its complications. Also the presence of the DNA of the* Porphyromonas gingivalis*, the bacteria intimately related to periodontitis in the periodontal pockets as well as in the atherosclerotic plaques of coronary and carotid arteries in patients with chronic periodontitis, who were hospitalized and underwent surgery because of vascular diseases, was demonstrated.

## 2. Material and Methods

The study population consisted of 91 patients (78 men and 13 women) treated in the Clinic of Cardiac Surgery or in the Clinic of Vascular, General and Transplantation Surgery, Wroctaw Medical University.

Group A consisted of 32 patients from 44 to 74 years of age with coronary artery disease and group B consisted of 31 patients from 46 to 84 years of age scheduled for carotid endarterectomy. Chronic periodontitis had been diagnosed in all these patients.

Group C was made up of 28 patients from 50 to 77 years treated in both the abovementioned clinics, who had been edentulous for at least 2 years before examination (Table 1).

All participants were fully informed about the procedures and informed consent was obtained from all patients.

The study protocol was approved by the ethical committee of Wroctaw Medical University.

Periodontal examination was performed by one trained and calibrated periodontist [MS]. Measurements of the approximal plaque index (API), bleeding on probing index (%BOP), and periodontal pocket depth (PD) at six sites per tooth using a manual, UNC-15 periodontal probe were subsequently recorded. Moderate periodontitis was diagnosed if at least one pocket ≥5 mm was present and severe periodontitis when at least one lesion ≥7 mm was found [15].

Bacteriological samples were collected from the 3 deepest periodontal pockets of each dentate patient. After drying the site and isolation from saliva, a sterile paper point was inserted into the pocket for 10 s, then transferred to a sterile Eppendorf tube, and sent to the laboratory.

The surgical procedures for carotid endarterectomy and coronary artery bypass graft surgery (CABG) were performed 1 week after periodontal examination. Atheromatous plaques from patients with carotid arteries stenosis were harvested during the surgery and were placed in a sterile tube with 10 mL of saline solution and frozen at −20°C.

In patients operated on with CABG there was no possibility to harvest atheromatous plaques, so during the surgery sterile paper points were inserted into the coronary vessel for 10 s and then immediately placed in sterile tubes and frozen at −20°C [16].

The laboratory tests were carried out in the Department of Forensic Medicine, Molecular Techniques Unit, Wroctaw Medical University.

For DNA extraction, 100 mg of atherosclerotic plaque was homogenized in 680 *μ*L 1x TEN buffer (0.1 M NaCl, 10 mM Tris-HCl pH 8.0, and 1 mM EDTA pH 8.0) using a bead beater at maximum speed for 20 s in three series. Then, the homogenized material was incubated for 16 hours at 55°C with 0.25 mg/mL proteinase K and 1.25% SDS. For purification of DNA, 1 mL of phenol : chloroform : isoamyl alcohol (25 : 24 : 1) was added and after centrifugation the aqueous phase was collected. DNA was precipitated with 99.8% ethanol and centrifuged. The DNA pellet was washed with 70% ethanol, dried, and dissolved in 100 *μ*L water.

In the case of swabs from periodontal pockets (groups A and B) and coronary arteries, DNA extraction was replaced by alkaline lysis with 0.2 M NaOH. After incubation for 5 min at 75°C, the samples were neutralized with 0.04 M Tris-HCl, pH 7.5. Solutions thus prepared were used as templates in PCR.

For detection of* Porphyromonas gingivalis* DNA specific primers were used as described by Slots et al. [17]: forward primer 5′-aggcagcttgccatactgcg and reverse primer 5′actgttagcaactaccgatgt. Amplification reactions were carried out in 25 *μ*L reactions with 0.2 *μ*M forward and reverse primers, 0.2 mM of each dNTPs, 2U DFS-Taq DNA polymerase (BIORON), and ≈50 ng template DNA. The thermal profile consisted of initial denaturation at 94°C for 2 min followed by 35 cycles of denaturation at 94°C for 10 s, annealing at 59°C for 20 s, and extension at 72°C for 1 min. The amplicons were visualized by electrophoresis using 1% agarose gel with 0.5 *μ*g/mL ethidium bromide. A Gene Ruler 100 bp DNA Ladder (Fermentas) was used as a molecular size standard. As a negative control water and NAOH/TRIS-HCl buffer were used. In case of positive control, we based on the reference strain of* Porphyromonas gingivalis* W83 after lysis and neutralisation procedures or DNA isolated from it.

Results were expressed as mean ± standard deviation (SD) for quantitative variables. The gathered data was analyzed statistically with Fisher's exact test.

## 3. Results and Discussion

The mean number of teeth in patients from group A was 14.1 and from group B was 12.7. All patients had poor oral hygiene (API value within the range 46–100%), and the mean value of this index in A and B groups was 88.6% and 87.7%, respectively. Despite the low level of oral hygiene, the mean bleeding index (BOP) was 38.5% in group A and 30.4% in patients from group B. The mean pocket probing depth was 3.36 mm in group A patients and 3.28 mm in group B (Table 2).

The DNA of* Porphyromonas gingivalis* was detected in 28 samples from periodontal pockets in group A patients (87.5%) and in 19 patients in group B (61.3%). There was no statistically significant association between these findings. Comparison between these two test groups of patients showed a difference in the prevalence of* Porphyromonas gingivalis* DNA in samples taken from vessels. Only 3 samples from group A were positive (9.4%), whereas DNA of* Porphyromonas gingivalis* was detected in 15 atheromatous plaques from patients from group B (48.4%). In group C consisting of edentulous patients,* Porphyromonas gingivalis* DNA was isolated in three samples (2 from patients with carotid artery stenosis and 1 treated because of the reduced permeability of coronary arteries) (Table 3). There was no relationship between the presence of the DNA of the test bacteria in periodontal pockets and atheromatous plaques/swabs of the blood vessels.

Currently, atherosclerosis is considered to be an inflammatory disease and not just a disease resulting from the accumulation of lipids in the vessel wall. An important element in prevention of atherosclerosis and its complications is an understanding of pathomechanisms leading to blood vessel wall damage.* Porphyromonas gingivalis*, the microorganism closely connected with chronic periodontitis, should be considered as one of the possible causes of eliciting general inflammation. With the use of PCR in several studies* Porphyromonas gingivalis* DNA was detected in atherosclerotic plaques of patients with chronic periodontitis. Toyofuku et al. [18] checked for the presence of bacterial DNA in samples obtained from 53 atherosclerosis patients.* Porphyromonas gingivalis* DNA was detected in 52% of arterial samples. Marcelino et al. [19] collected and analyzed DNA of periodontal pathogens in atheromatous plaques from 28 patients. Samples were positive for all bacteria except for* Fusobacterium nucleatum*.* Porphyromonas gingivalis* DNA was present in 50% of samples. Ishihara et al. [20] obtained samples of atheromatous plaques from the coronary arteries of 51 patients. As in previous studies, the PCR method was used. In 21.6% of the samples the DNA of* Porphyromonas gingivalis* was present. Similar results were published by Mahendra et al. [21], who examined 51 samples of atheromas from the coronary arteries of patients with chronic periodontitis.* Porphyromonas gingivalis* DNA was detected in 45.1% of samples. Equally high positive results (53.8%) for the presence of* Porphyromonas gingivalis* DNA have been obtained by Brazilian scientists. They evaluated atherosclerotic coronary arteries of 39 patients with chronic periodontitis [22].

The results of present study confirm the findings of the cited authors.* Porphyromonas gingivalis* DNA was detected in 21 samples from vessels. The majority of positive results (*n* = 15) originated from patients with carotid atherosclerosis from whom atherosclerotic plaques were taken to be tested. Since in patients operated on because of coronary atherosclerosis there was no possibility to harvest atheromatous plaques due to operation protocol, sterile paper points were tested after a 10-second contact with the vessel plaque. The percentage of positive results in this group was significantly lower and amounted to 9.4% (3 individuals). It seems that such a significant discrepancy in the detection of bacterial DNA may result from differences in the method of material collection, as* Porphyromonas gingivalis* has the ability to penetrate into the endothelial cells. Thus, there is a much higher probability of isolation of this pathogen from the complete atherosclerotic plaque. By contrast, despite the presence of these bacteria in endothelial cells, detection of their DNA in the filtrate obtained from the paper point contact with stable plaque covered with a layer of fiber may be much more difficult or not possible. However, it is interesting to note that in other published studies using a similar method a higher percentage of positive results was achieved. Zaremba et al. [16] checked samples from 20 patients with chronic periodontitis who were scheduled for coronary artery bypass grafting (CABG) because of coronary artery obstruction. They reported the presence of* Porphyromonas gingivalis* DNA in the filtrate of atherosclerotic plaques in 10 cases, which accounted for 50% of patients.

In contrast to the present study and the results of other authors cited above, Cairo et al. [23], when examining 40 samples of atherosclerotic plaques (obtained after carotid endarterectomy) by PCR, did not detect the presence of any periodontal pathogenic bacteria. Aimetti et al. [24] did not isolate any periopathogens in samples taken from atherosclerotic carotid arteries of patients with periodontitis. Padilla et al. [25] detected the DNA of* Aggregatibacter actinomycetemcomitans* (the authors checked the presence of four periopathogens) in only 2 out of 12 atherosclerotic plaques from patients who had periodontitis diagnosed and were operated on because of artery stenosis (carotid, popliteal, tibial, and femoral arteries). These authors suggest that such discrepancies in the results from different studies may be associated with the varying methods of laboratory analysis. In studies using a PCR differences may result from various methods of DNA extraction, use of unlike sequences of primers, and different reaction conditions. An example of such situation is a multicenter PCR comparison trial published by Apfalter et al. [26], who sent the same atheromatous plaques to nine different laboratories for the detection of* Chlamydia pneumoniae* DNA. Depending on the different test methods the positivity rate varied between 0 and 60%. These variations resulted from the different conditions and testing methods, since laboratories were free to choose the method.

Kozarov et al. [27] assumed that the presence of bacterial DNA in vessel walls does not prove the presence of live bacteria which are able to invade cells and to induce inflammation. Therefore, they conducted a study using quantitative PCR and then they stained the examined tissue. In samples of atheromatous plaques live bacteria* Porphyromonas gingivalis* and* Aggregatibacter actinomycetemcomitans* were present inside the endothelial cells. This study proved for the first time that detected bacterial DNA is derived from live bacterial cells, because only live microorganisms are able to penetrate into cells other than phagocytes [28].

The question also arises as to whether there is a correlation between the presence of* Porphyromonas gingivalis* in periodontal pockets and in the atherosclerotic plaques. In the present study there was no such relationship. In groups of dentate patients (A + B) the simultaneous presence of bacterial DNA in the samples from the periodontal pockets and in samples taken from blood vessels was confirmed in 19%, while in group B alone in 29%. Nevertheless, there was no statistically significant relationship. Similar results were published by Zhang et al. [29], who detected the simultaneous presence of* Porphyromonas gingivalis* DNA in the atheromatous plaques and periodontal pockets of 10 out of 51 patients (19.6%). In a group of 20 patients Zaremba et al. [16] marked the presence of* Porphyromonas gingivalis* DNA in both locations (periodontal pockets and the filtrate from atherosclerotic coronary arteries) in 5 patients (25%). However, in the studies conducted by Mahendra et al. [21] the rate was 39.22%. Ishihara et al. [20] reported the presence of* Porphyromonas gingivalis* 16S rRNA in samples of coronary atherosclerotic plaques obtained from 51 patients, and this was significantly positively correlated with the presence of this microorganism in periodontal pockets (*P* < 0.01). The fact that bacterial DNA is rarely isolated from atherosclerotic plaques compared to periodontal pockets and that usually there is no positive correlation between the simultaneous occurrences of periopathogens in both study locations is explained by the authors as being a result of the influence of the organism's defense system to the elimination of these bacteria, either through the cells of the first line of defense or infiltration of granulocytes, as well as a specific defense by running the humoral and cellular responses. This is not a convenient environment for their growth and reproduction. What is more in 3 cases* Porphyromonas gingivalis* DNA was present in samples from edentulous patients. It is worth emphasizing that edentulous patients had no periodontium, so there have been no periopathogens present in oral cavity for at least 24 preceding months.

In our study, in 6 patients no DNA of* Porphyromonas gingivalis* was detected in periodontal pockets but it was determined in samples from blood vessels. Such a situation can be explained by the fact that in chronic periodontitis short periods of exacerbation of inflammation and prolonged periods of remission are observed. In addition, most patients in both study groups were elderly and they had lost some teeth (the “elderly” is defined as ≥65 years according to the contractual age limit adopted by most developed countries). As one of the main causes of tooth loss patients reported “mobility” of the teeth which can be linked to an advanced ongoing inflammatory-destructive state in periodontal tissues. It is likely that in deep periodontal pockets of teeth with increased mobility the bacteria of “red complex,” including* Porphyromonas gingivalis*, were present. During this period there may have been an invasion of bacteria into the circulatory system; then the teeth were lost. Periodontal pockets of the teeth present in the oral cavity, from which samples for testing were taken, may be shallower and therefore not colonized by the most dangerous strains of strictly anaerobic bacteria.

## 4. Conclusions

In summary, we can conclude that it is possible that recognized periodontal pathogens present in subgingival oral biofilm may find a way to reach arteries, so ongoing periodontitis may be one of the factors affecting patients at risk of atherosclerosis and related diseases. For this reason, periodontal care should be treated as a necessity for all patients at risk of vascular and cardiac disorders.

## Figures and Tables

**Table 1:** Characteristics of groups.

Characteristic	Group A (*N* = 32) Patients with coronary artery disease	Group B (*N* = 31) Patients scheduled for carotid endarterectomy	Group C (*N* = 28) Patients edentulous
Age (years)	59.3 (SD = 7.36)	63.9 (SD = 9.31)	65.1 (SD = ±7.2)
Gender (male/female)	31/1	24/7	23/5
Smoking (current and former smokers who stopped <5 years before entering the study)	15	19	17

**Table 2:** Mean values ± SD of periodontal indices in groups A and B.

Variable	Group A (*N* = 32) Patients with coronary artery disease	Group B (*N* = 31) Patients scheduled for carotid endarterectomy
Number of teeth	14.1 (SD = 5.8)	12.7 (SD = 6.1)
Mean API^*∗*^	88.6% (SD = 13.9)	87.7% (SD = 14.1)
Mean BOP^*∗∗*^	38.5% (SD = 14.4)	30.4% (SD = 19.8)
Mean probing depth	3.36 mm (SD = 0.77)	3.28 mm (SD = 0.93)

^*∗*^API: approximal plaque index.

^*∗∗*^BOP: bleeding on probing.

**Table 3:** Positive results of polymerase chain reaction detection in samples from vessels and from periodontal pockets.

	Group A (*N* = 32) Patients with coronary artery disease	Group B (*N* = 31) Patients scheduled for carotid endarterectomy	Group C (*N* = 28) Patients edentulous
Periodontal pockets	28	19	NA
Atheromatous plaques/smear from coronary arteries	3	15	3
